# Differentiation of placenta percreta through MRI features and diffusion-weighted magnetic resonance imaging

**DOI:** 10.1186/s13244-023-01448-z

**Published:** 2023-05-24

**Authors:** Hang Li, Tao Lu, Mou Li, Yishuang Wang, Feng Zhang, Yi Yuan, Meilin Zhu, Xinyi Zhao

**Affiliations:** Department of Radiology, Sichuan Provincial People’s Hospital, University of Electronic Science and Technology of China, 32 West Second Section, First Ring Road, Chengdu, 610072 China

**Keywords:** Placenta accreta spectrum disorders, Diffusion-weighted MRI, Intravoxel incoherent motion, Diffusion kurtosis imaging

## Abstract

**Objectives:**

To identify whether parameters measured from diffusion kurtosis and intravoxel incoherent motion help diagnose placenta percreta.

**Methods:**

We retrospectively enrolled 75 patients with PAS disorders including 13 patients with placenta percreta and 40 patients without PAS disorders. Each patients underwent diffusion-weighted imaging (DWI), intravoxel incoherent motion (IVIM), and diffusion kurtosis imaging (DKI). The apparent diffusion coefficient (ADC), perfusion fraction (*f*), pure diffusion coefficient (*D*), pseudo-diffusion coefficient (D*), mean diffusion kurtosis (MK) and mean diffusion coefficient (MD) were measured by the volumetric analysis and compared. MRI features were also analyzed and compared. The receiver operating characteristic (ROC) curve and logistic regression analysis were used to evaluate the diagnostic efficiency of different diffusion parameters and MRI features for distinguishing placental percreta.

**Results:**

D* was an independent risk factor from DWI for predicting placenta percreta with sensitivity of 73% and specificity of 76%. Focal exophytic mass remained as independent risk factor from MRI features for predicting placenta percreta with sensitivity of 72.7% and specificity of 88.1%. When the two risk factors were combined together, the AUC was the highest, 0.880 (95% CI 0.8–0.96).

**Conclusion:**

D* and focal exophytic mass were associated with placenta percreta. A combination of the 2 risk factors can be used to predict placenta percreta.

**Critical relevance statement:**

A combination of D* and focal exophytic mass can be used to differentiate placenta percreta.

## Key points

1. D* and focal exophytic mass were associated with placenta percreta.
2. DWI parameters help quantify PAS.
3. Placenta percreta can be differentiated with combination of risk factors.

## Introduction

Placenta accreta spectrum (PAS) disorders are the abnormal trophoblast invasion into the myometrium where the Nitabuch’s layer is disrupted [1]. The depth of villous tissue invasiveness into the myometrium is different, and placenta percreta is the most aggressive form when the placental villi penetrate through the entire myometrium and even to the surrounding organs. As the abnormal placenta is strongly attached to the myometrium and/or the extrauterine tissues, the risk of postpartum hemorrhage increases when any attempt to remove the abnormal placenta. Placenta percreta is life-threatening and is also associated with other serious maternal complication including local organ damage, peripartum hysterectomy and even death [2, 3]. The maternal morbidity was 3 times greater in patients with placenta percreta than those with accreta/increta [4]. Accurate and timely prenatal diagnosis of placenta percreta allows time for multidisciplinary team work including preparation for blood transfusion, hysterectomy and ICU (intensive care unit) stay to improve maternal outcome.

Currently, MRI (magnetic resonance imaging) is used as a complementary modality to ultrasound in diagnosing PAS. Although the accuracy of MRI and US (ultrasonography) is similar, MRI is better in depicting the topography, depth and extension of placental invasion, particularly in detection of parametrium, uterine ligament and bladder extension. Placental bulge, bladder wall interruption, bladder tenting, bladder vessel sign, serosa vessel sign and parametrial vessel sign have all been reported to be associated with placenta percreta [5, 6]. However, a recent study suggests experience plays a significant role in accurately interpreting PAS-related MRI findings. Interpreting MRI findings is considered challenging even for the most experienced radiologist [7].

Diffusion-weighted magnetic resonance imaging is a functional MRI technique that measures water molecular movement within the tissue. IVIM (intravoxel incoherent motion) is another DWI technique that measures both water molecular diffusion and blood microcirculation in the tissue using enough low *b*-values [8]. DKI (diffusion kurtosis imaging) is a polynomial DWI model that measures the deviation of water diffusion from a homogeneous, unrestricted and free distribution using more larger *b*-values [9]. Using IVIM and DKI, our previous studies showed *D* mean and *D* max can be used to discriminate PAS disorders, *D* mean was also significantly higher in patients with placenta increta and percreta [10, 11]. However, our previous studies did not focus on the value of IVIM and DKI for distinguishing placenta percreta and did not investigate if IVIM and DKI can help improve the diagnosis of placenta percreta. Therefore, the purpose of our study was to explore the diagnostic accuracy of IVIM and DKI parameters for differentiation of placenta percreta, and to compare the diagnostic performance of MRI features and DWI parameters.

## Materials and methods

**Fig. 1:**
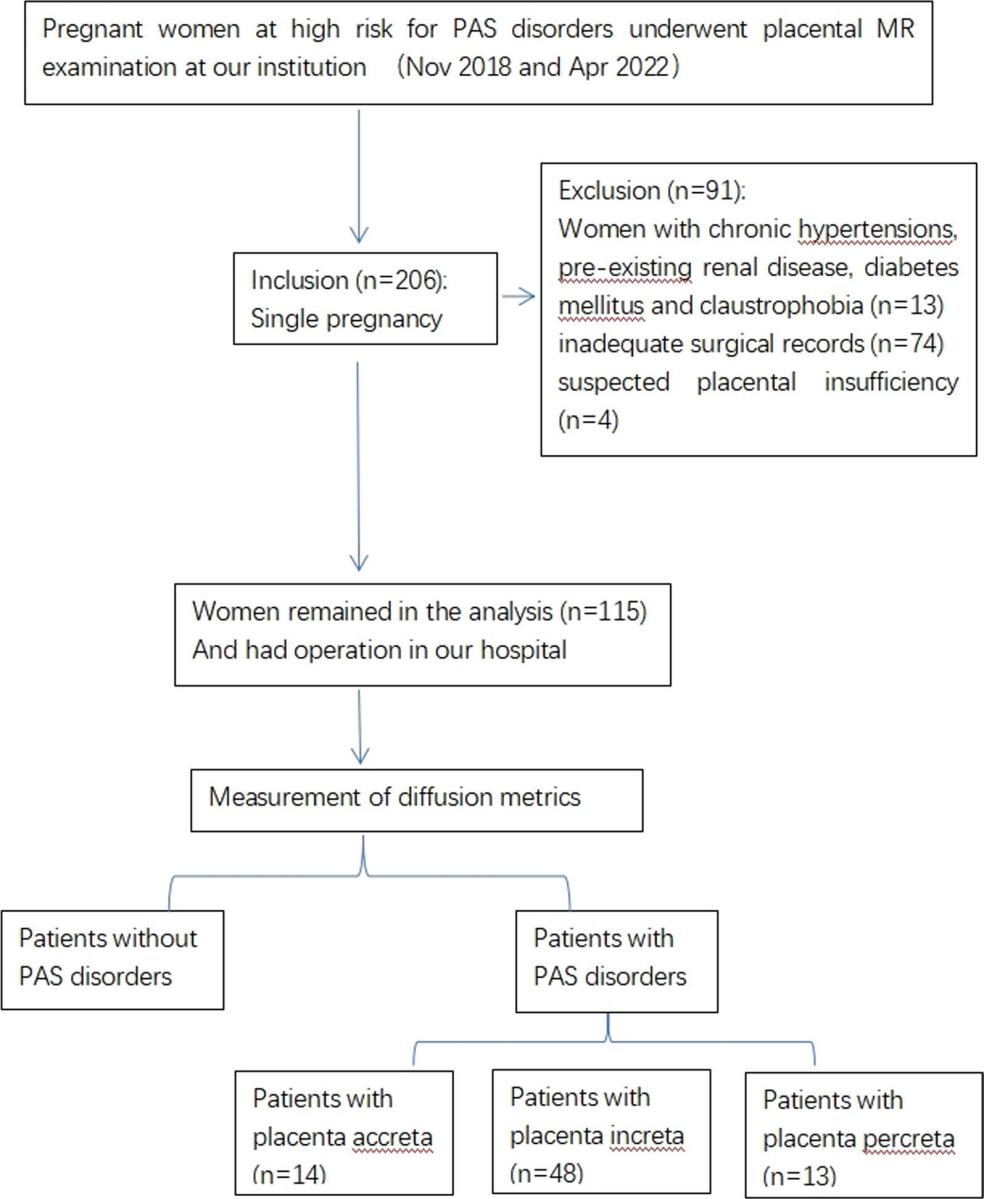
Study design flowchart

Our institutional review board (IRB) approved this study, and we obtained written informed consent from each female participant. From November 2018 to April 2022, 206 patients underwent placental MRI including a DWI sequence. The inclusion criteria were: (1) suspected PAS disorders based on clinical risk factors or uncertain ultrasound (US) results, (2) singleton pregnancy, and (3) fetal development coinciding with gestational age. Patients were excluded for the following reasons: (1) presence of any maternal pathology, (2) inadequate surgical records, (3) suspected placental insufficiency, or (4) severe artifacts on MRI images (Fig. 1).

### Clinical characteristic analysis

Information on maternal age, gravidity, parity, number of previous CDs, and number of abortions, gestational age at examination and gestational age at delivery was recorded from the clinical records of the patients.

### MR imaging protocols

All MR images were acquired at a 1.5T MR scanner (Aera, Siemens Healthineers, Erlangen, Germany) using a 16-channel body matrix coil. The following sequences with current scanning parameters were included: (1) axial, coronal, and sagittal half-Fourier acquisition single-shot turbo spin echo (HASTE): field of view (FOV) 420 × 80 mm, 5-mm-thick section, 20% gap, matrix 272 × 320, scan duration 50 s; (2) axial, coronal and sagittal true fast imaging in steady-state precession (TRUFISP): FOV of 420 × 80 mm, 5-mm-thick section, 30% gap, matrix 234 × 384, and a scan duration of 48 s; (3) 3D-volumetric interpolated breath-hold examination (3D-VIBE): FOV 400 mm, 5-mm-thick section, 20% gap, matrix 180 × 320, scan duration 8 s; (4) Diffusion-weighted imaging: FOV 390 mm, 5-mm-thick section, matrix 192 × 120, parallel imaging acceleration factor 2, *b* values ranging from 0 to 1600 s/mm^2^ (*b* = 0, 50, 100, 150, 200, 400, 600, 800, 1000, 1200, and 1600 s/mm^2^), scan duration 7 min 29 s.

### Imaging analysis

For standard monoexponential DWI model, the image data of 2 *b*-values (0 and 1000 s/mm^2^) were used to generate ADC map:

$$S_{b} /S_{0} = \exp ( - b \times {\text{ADC}}),$$

where *S*_*b*_ and *S*_*0*_ are the signal intensities in the diffusion gradient factors of *b* and 0, respectively. ADC is the apparent diffusion coefficient.

For DKI model, the image data of six *b*-values (*b* = 0, 400, 800, 1000, 1200, and 1600 s/mm^2^) were used to generate MD and MK maps [12, 13]:

$$S_{b} /S_{0} = \exp ( - b \times {\text{MD}} + b2\,{\text{MD}}2 \times {\text{MK}}/6),$$

where *S*_*b*_ and *S*_0_ are the signal intensities acquired with the diffusion gradient factors of *b* and 0, respectively. MD is the mean diffusivity representing the corrected ADC, and MK is the diffusion kurtosis.

For the IVIM analysis, a bi-exponential model was fitted using eight *b*-values (*b* = 0, 50, 100, 150, 200, 400, 600, and 800 s/mm^2^) [14, 15]:

$$S_{b} /S_{0} = (1 - f)\,\exp \,( - b \times D) + f\,\exp \,[ - b \, \times (D + D*)],$$

where *S*_*b*_ and *S*_0_ are the signal intensities in the diffusion gradient factors of *b* and 0, respectively, *f* is the perfusion fraction, *D* is the diffusion coefficient, and D* is the pseudo-diffusion coefficient.

**Fig. 2:**
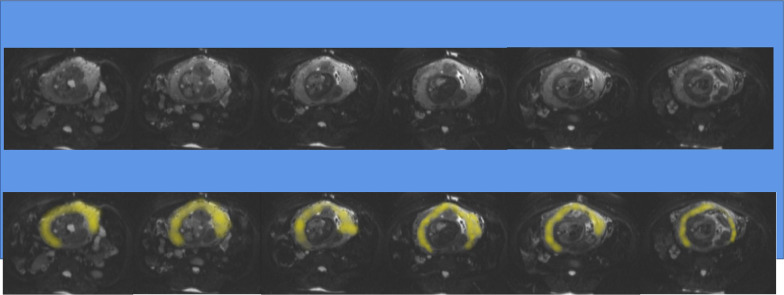
Schematic illustration of ROI delineation of the whole placenta measurement

**Fig. 3:**
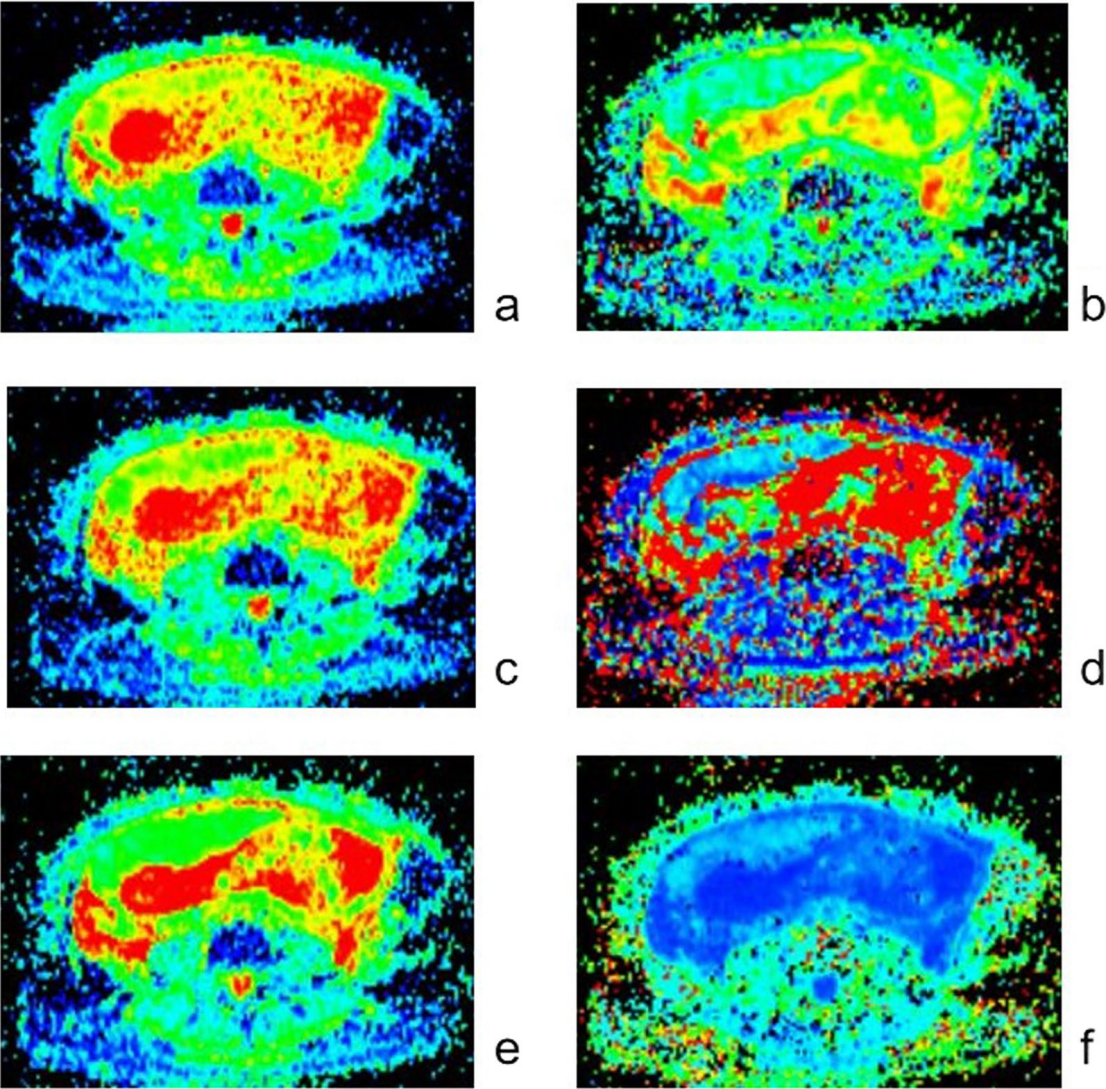
The diffusion parameter maps of ADC (**a**), *f* (**b**), *D* (**c**), D* (**d**), MD (**e**) and MK (**f**)

All ROIs were drawn in all axial slices from *b* = 0 s/mm^2^ images including the whole placenta and then, copied to all diffusion parameter maps (Fig. 2). ROIs were drawn independently by 1 radiologist with 3 years of experience in obstetric imaging using research software IMAgenGINE (Vusion Tech) [16]. The ADC, MD, MK, *D*, D*, and *f* maps were automatically produced, and the parameters were automatically calculated (Fig. 3).

All MRI images were reviewed by 2 radiologists with 5 and 10 years of experience in obstetric imaging; they reached a consensus in cases of disagreement. The readers were blind to ultrasound diagnosis, surgical and pathological findings and were asked to review the MR images and record the presence of any of the following features including T2 dark bands, placental heterogeneity, abnormal intraplacental vascularity, placental cervical protrusion sign, focal exophytic mass, placental recess, placental bulge, abnormal vascularization of the placental bed, myometrial interruption, bladder tenting, bladder vessel sign and parametrial vessel sign [5, 17–19].

## Reference standard

The diagnosis of PAS disorders was primarily based on intraoperative gross findings. Surgical evidence of placenta percreta included disruption of the outer myometrial layer and visualization of placental tissue invading the uterine serosa and surrounding organs, including the broad ligament, vaginal wall, and bladder visually. Placenta increta was diagnosed when the placenta did not separate after 20 min despite active management, resulting in a difficult manual piecemeal removal of the placenta and heavy bleeding from the implantation site during the 3rd stage of labor. Placenta accreta was diagnosed when the placenta firmly adhered to the endometrium with uncontrollable bleeding at the time of abruption. Pathological examination of the uterine specimens in hysterectomy cases or tissue samples obtained from invasive area were used to confirm surgical diagnosis.

### Statistical analysis

Continuous variables with a normal or nonnormal distribution were expressed as means ± standard deviation (SD) or median (range), respectively, and categorical variables were expressed as absolute numbers (proportions, %).The Mann–Whitney *U*-test and *χ*2 test were used to compare the clinical features of patients with and without PAS disorders. Kruskal–Wallis *H*-test was also used to compare the difference in DWI parameters between patients with placenta accreta, increta, percreta and normal placentas. A multivariate logistic regression analysis with a stepwise forward procedure was used to determine the most significant risk factors for predicting placenta percreta. In addition, receiver operating characteristics (ROC) analyses were performed to evaluate the diagnostic performance of significant parameters and to estimate the discriminative ability of MRI features. The significant DWI parameters showing the highest Youden index were included for the differentiation. *p* values < 0.05 were considered statistically significant. All analyses were performed using SPSS 21.0 (IBM Inc).

## Results

A total of 115 patients were retrospectively included in the study. The mean maternal age was 32 years ranging between 22 and 45 years. The mean gestational age at examination was 31 weeks, ranging between 22 and 38 weeks. The clinical characteristics of the 2 groups are shown in Table 1. Of the 115 patients, 75 (66.09%) were diagnosed as PAS disorders, including 14 of placenta accreta, 48 of placenta increta and 13 of placenta percreta.

**Table 1:** Maternal characteristics in the study groups

	Patients without PAS disorders	Patients with PAS disorders	*p* value
Number	40	75	
Age (years)	29.15 ± 4.11	32.65 ± 4.35	0.000
Less than 35	35 (87.5%)	51 (68%)	0.022
35 or older	5 (12.5%)	24 (32%)	
Gestational age	31 (4.5)	31 (5)	0.864
At examination (weeks)			
Gestational age	38 (2.75)	36 (2)	0.000
At the time of delivery (weeks)			
Previous caesarean			0.067
Section			
Yes	20 (50%)	54 (72%)	
No	20 (50%)	21 (28%)	
Number of previous caesarean			0.167
Section			
0	20 (50%)	24 (32%)	
1	17 (42.5%)	43 (57.33%)	
2 or more	3 (7.5%)	8 (10.67%)	
Previous uterine			0.052
Dilation and curettage			
Yes	26 (65%)	61 (81.33%)	
No	14 (35%)	14 (18.67%)	
Number of previous uterine			0.174
Dilation and curettage			
0	14 (35%)	11 (14.67%)	
1	11 (27.5%)	23 (30.67%)	
2 or more	15 (37.5%)	41 (54.67%)	
Placenta previa			0.000
Yes	15 (37.5%)	72 (96%)	
No	25 (62.5%)	3 (4%)	

Patients with PAS disorders were older compared to those without PAS disorders (*p* < 0.05). When compared to patients without PAS disorders, patients with PAS disorders were more likely to have prior CS and placenta previa (*p* < 0.05). The number of prior dilation and curettage, gravidity and parity did not differ between patients with and without PAS disorder (*p* > 0.05).

### Performance of DWI parameters

**Fig. 4:**
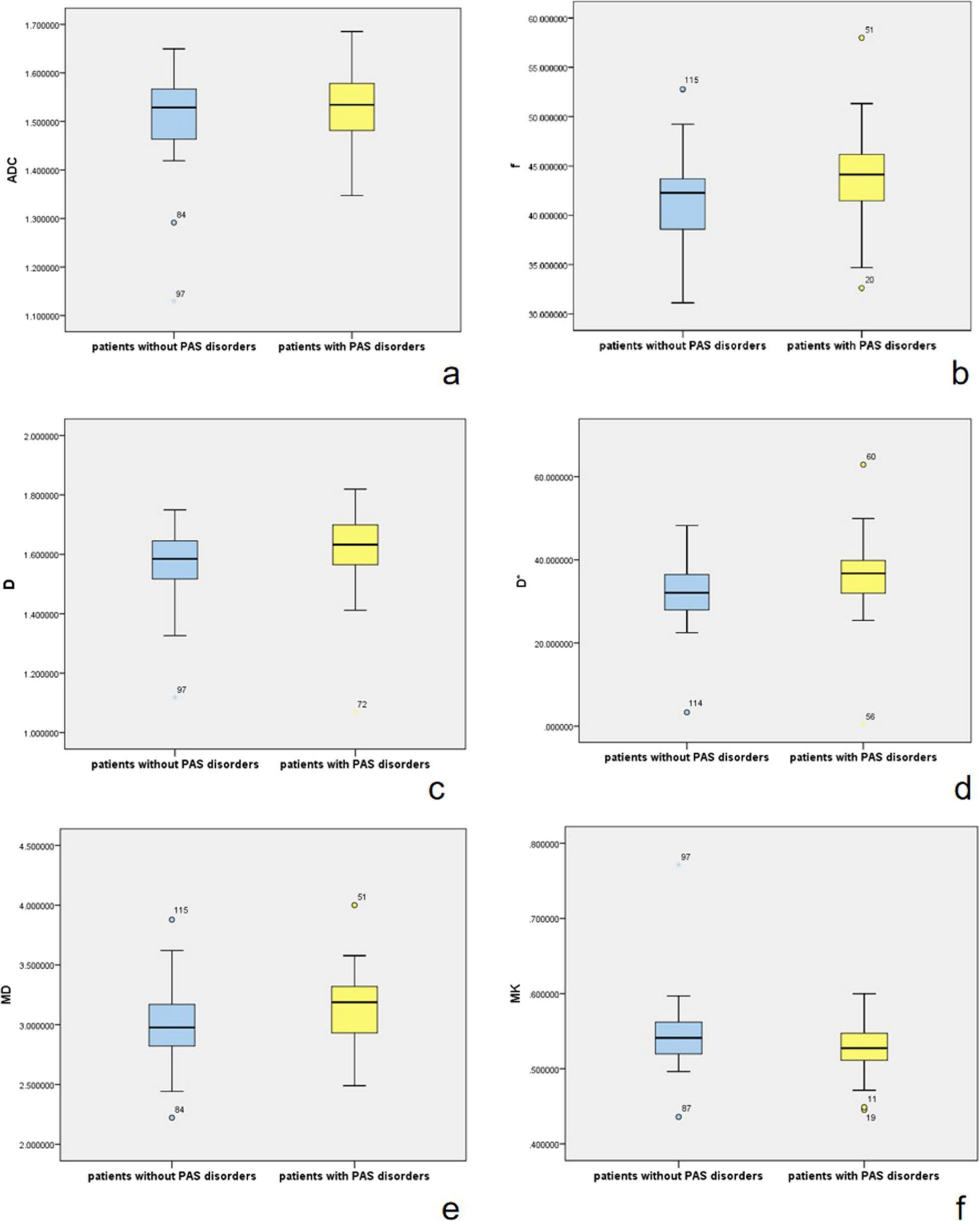
Box and whisker plots of ADC, *D*, D*, *f*, MD, and MK for patients with and without PAS disorders (**a**–**f**). The plots show that *f*, *D*, D*, and MD are significantly higher (**d**–**f**) in patients with PAS disorders

**Fig. 5:**
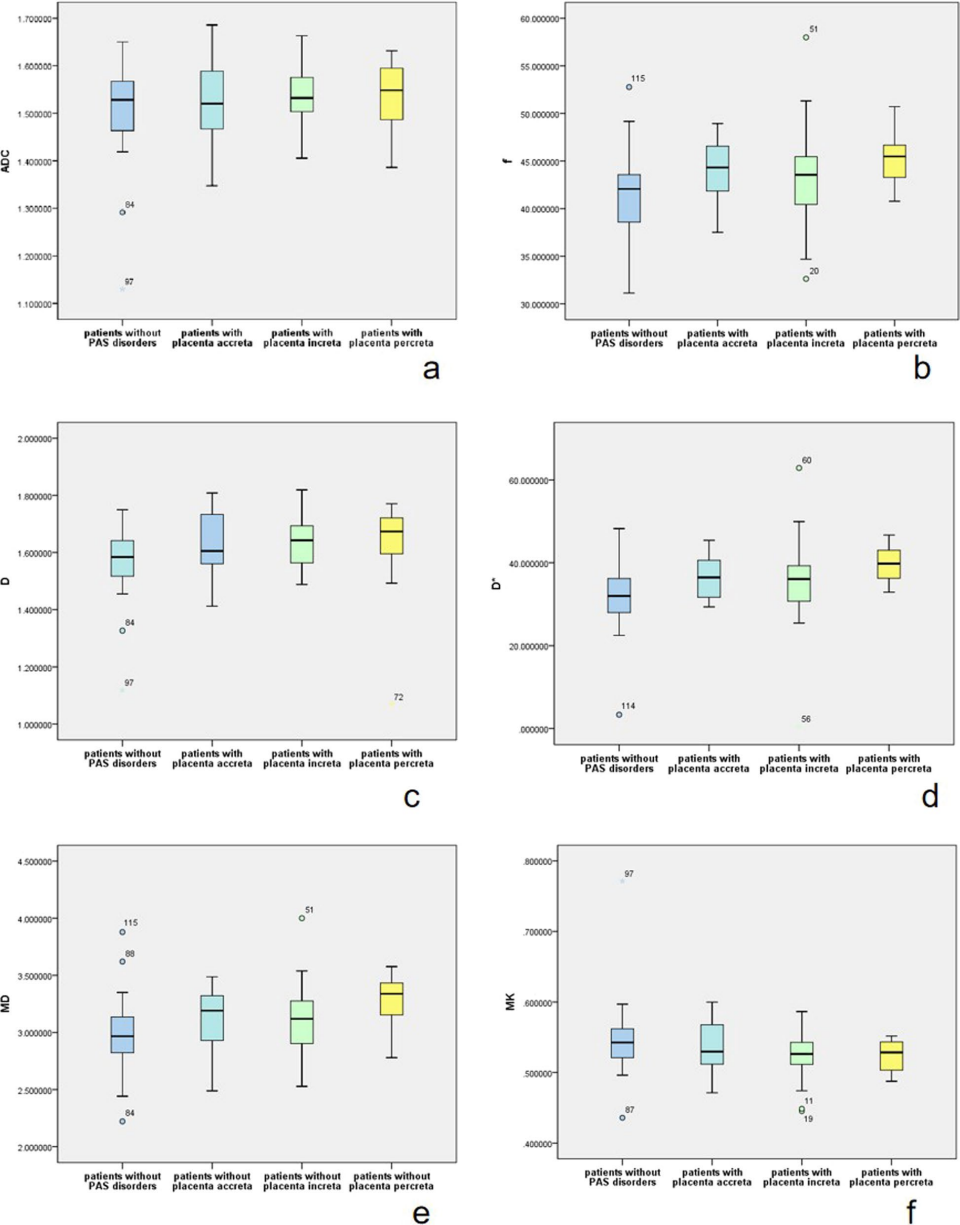
Box and whisker plots of ADC, *D*, D*, *f*, MD, and MK for patients with and without placenta percreta (**a**–**f**). The plots show that *f*, D* and MD are significantly higher (**d**–**f**) in patients with placenta percreta

**Fig. 6:**
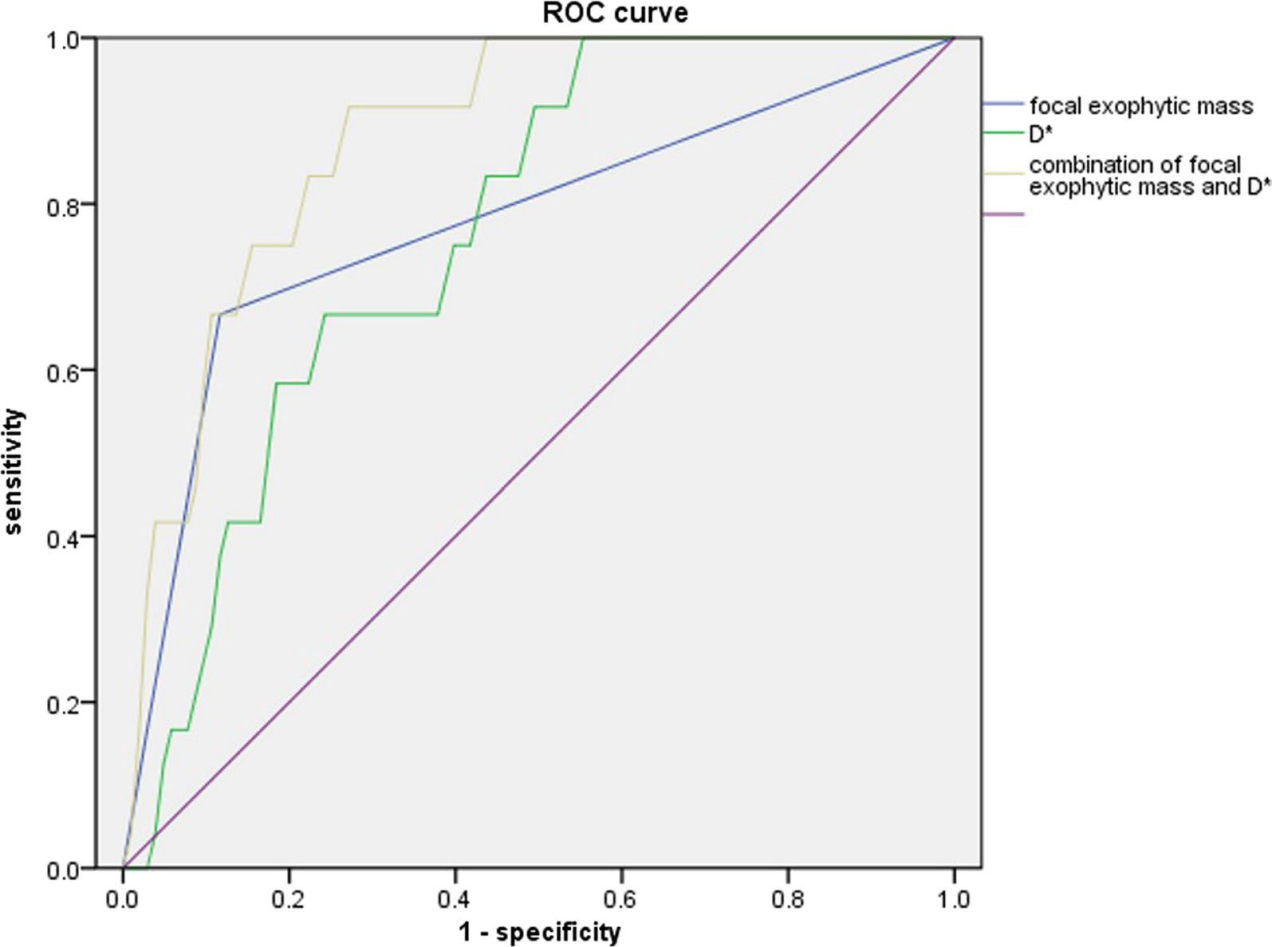
ROC curves for predicting patients with placenta percreta. A combination of number of D* and focal exophytic mass shows the best overall performance

DWI parameter comparisons showed that *f*, *D*, D*, and MD were significantly higher (*p* = 0.011, 0.015, 0.001 and 0.01, respectively) in patients with PAS disorders (Table 2, Fig. 4). Multiple comparisons showed *f*, D* and MD were significantly higher (*p* = 0.015, 0.001 and 0.008, respectively) in patients with placenta percreta than those in patients without PAS disorders (Table 3 and Fig. 5). On multiple logistic regression analysis, D* was an independent risk factor in predicting placenta percreta (*p* = 0.018). For predicting placenta percreta, D* demonstrated an AUC of 0.778 (95%CI 0.666–0.890) with a cutoff value of 39.07 × 10^−3^mm^2^/s with Youden's index of 0.49 sensitivity of 73% and, specificity of 76% (Fig. 6).

**Table 2:** Comparison of DWI parameters between patients with and without PAS disorders (n = 115)

Parameters	Patients without PAS disorders	Patients with PAS disorders	*p* value
Standard DWI parameters			
ADC mean (× 10^−3^ mm^2^/s)	1.529 (0.11)	1.53 (0.10)	0.360
DKI parameters			
MD mean (× 10^−3^ mm^2^/s)	2.98 (0.35)	3.19 (0.39)	0.01
MK mean	0.54 (0.04)	0.53 (0.04)	0.061
IVIM parameters			
*f* mean (%)	42.27 (5.15%)	44.12 (5.23)	0.011
*D* mean (× 10^−3^ mm^2^/s)	1.58 (0.13)	1.63 (0.14)	0.015
D* mean (× 10^−3^ mm^2^/s)	32.07 (8.66)	36.75 (7.91)	0.001

**Table 3:** Comparison of DWI parameters between patients with different grades of PAS disorders (n = 115)

Parameters	Patients without PAS disorders	Patients with placenta accreta	Patients with placenta increta	Patients with placenta percreta	*p* value
Standard DWI parameters					
ADC mean (× 10^−3^ mm^2^/s)	1.53 (0.11)	1.52 (0.14)	1.53 (0.08)	1.55 (0.12)	0.754
DKI parameters					
MD mean (× 10^−3^ mm^2^/s)	2.97 (0.34)	3.19 (0.43)	3.12 (0.39)	3.34 (0.33)	0.008
MK mean	0.54 (0.04)	0.53 (0.06)	0.53 (0.03)	0.53 (0.04)	0.195
IVIM parameters					
*f* mean (%)	42.07 (5.09)	44.32 (5.13)	43.55 (5.31)	45.48 (3.91)	0.015
*D* mean (× 10^−3^ mm^2^/s)	1.58 (0.13)	1.61 (0.18)	1.64 (0.13)	1.67 (0.14)	0.048
D* mean (× 10^−3^ mm^2^/s)	31.95 (8.26)	36.44 (9.20)	36.07 (8.58)	39.78 (7.26)	0.001

### Performance of MRI features

Table 4 presents the diagnostic accuracy of different MRI features. T2 dark bands, placental heterogeneity, focal exophytic mass, abnormal vascularization of the placental bed, myometrial interruption, bladder tenting and parametrial vessel sign were associated with placenta percreta. On multiple logistic regression analysis, focal exophytic mass remained as independent risk factor for predicting placenta percreta (*p* = 0.001). Focal exophytic mass demonstrated an AUC of 0.804 with sensitivity of 72.7% and specificity of 88.1% (Fig. 6).

**Table 4:** Diagnostic performances of different features for association with placenta percreta (n = 115)

MRI features	No (%) of patients		Sensitivity (%)	Specificity (%)	PPV (%)	NPV (%)	AUC	*p*
	Patients without placenta percreta	Patients with placenta percreta						
T2 dark bands			72.7	70.3	71	72.29	0.715	0.002
No	72 (69.90)	3 (25)						
Yes	31 (30.10)	9 (75)						
Placental heterogeneity			45.5	84.2	74.23	60.71	64.8	0.016
No	86 (83.50)	6 (50)						
Yes	17 (16.50)	6 (50)						
Abnormal intraplacental vascularity			58.3	54.4	56.11	56.55	56.4	0.404
No	56 (54.37)	5 (41.67)						
Yes	47 (45.63)	7 (58.33)						
Placental cervical protrusion sign			25	90.3	72.05	54.63	57.6	0.113
No	93 (90.29)	9 (75)						
Yes	10 (9.71)	3 (25)						
Focal exophytic mass			72.7	88.1	85.93	76.34	80.4	0.000
No	91 (88.35)	4 (33.33)						
Yes	12 (11.65)	8 (66.67)						
Placental recess			16.7	96.1	81.07	53.57	56.4	0.059
No	99 (96.12)	10 (83.33)						
Yes	4 (3.88)	2 (16.67)						
Placental bulge			33.3	86.4	71	16.94	59.9	0.075
No	89 (86.41)	8 (66.67)						
Yes	14 (13.59)	4 (33.33)						
Abnormal vascularization of the placental bed			45.5	89.1	90.28	62.05	67.3	0.006
No	91 (88.35)	7 (58.33)						
Yes	12 (11.65)	5 (41.67)						
Myometrial interruption			54.5	77.2	70.50	62.92	65.9	0.039
No	79 (76.70)	6 (50)						
Yes	24 (23.30)	6 (50)						
Bladder tenting			18.2	100	100	55.01	59.1	0.010
No	103 (100)	10 (83.33)						
Yes	0 (0)	2 (16.67)						
Bladder vessel sign			8.3	100	100	52.6	54.2	0.104
No	103 (100)	11 (91.67)						
Yes	0 (0)	1 (8.33)						
Parametrial vessel sign			36.4	100	100	61.12	68.2	0.000
No	103 (100)	7 (58.33)						
Yes	0 (0)	5 (41.67)						

### Performance of combination of DWI parameters and MRI features

**Fig. 7:**
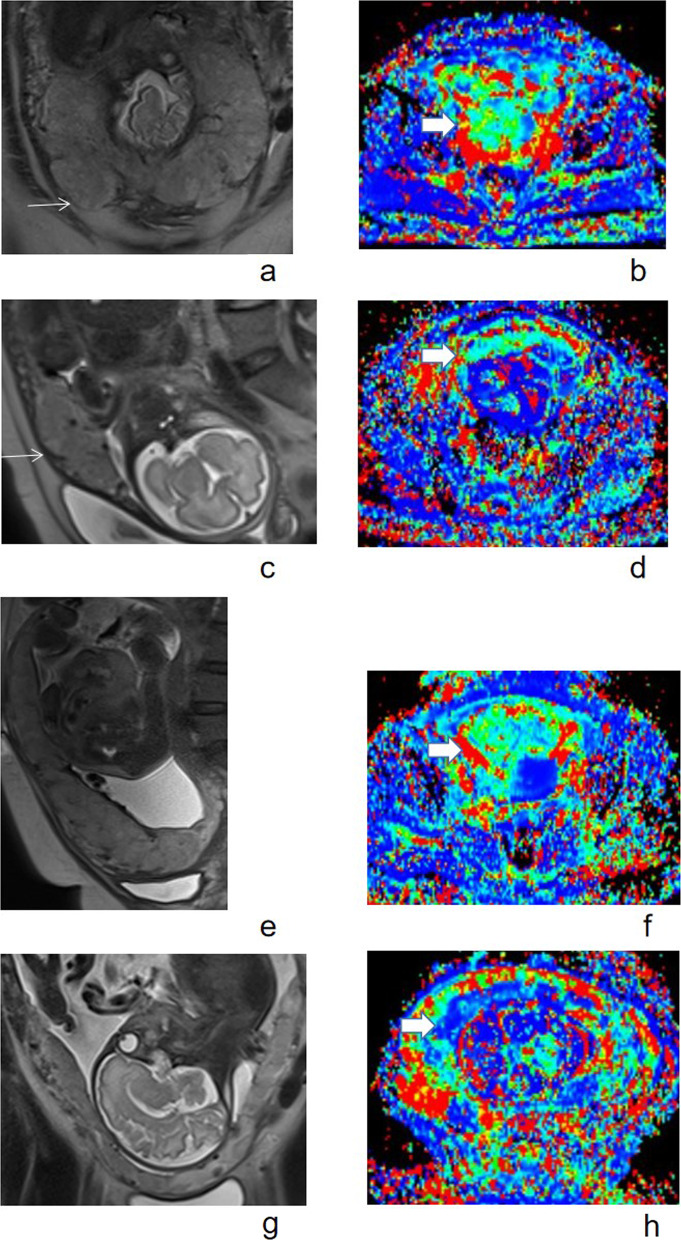
Illustration of D* and focal exophytic mass in patients with placenta percreta, increta and accreta. Figure 6a was a coronal HASTE image showing a 39-year-old woman with placenta previa and percreta. Focal exophytic mass can be seen (white arrow); Fig. 6b was D* map of the placenta with D* of 49.33 × 10^−3^ mm^2^/s. Figure 6c was a sagittal HASTE image showing a 30-year-old woman with placenta percreta. Focal exophytic mass can be seen (white arrow), Fig. 6d was D* map of the placenta with D* of 37.95 × 10^–3^ mm^2^/s. Figure 6e was a sagittal HASTE image showing a 40-year-old woman with placenta previa and increta. Focal exophytic mass cannot be seen. Figure 6f was D* map of the placenta with D* of 37.36 × 10^–3^ mm^2^/s. Figure 6g was a coronal HASTE image showing a 25-year-old woman with placenta previa and accreta. Focal exophytic mass cannot be seen. Figure 6h was D* map of the placenta with D* of 32.15 × 10^–3^ mm^2^/s

We combined D* and focal exophytic mass for predicting placenta percreta, producing the sensitivity of 92%, specificity of 74%, and AUC of 0.880 (95% CI 0.8–0.96) (Figs. 6, 7).

## Discussion

Our results showed D* and focal exophytic mass were independent risk factors from DWI parameters and MRI features, respectively, in predicting placenta percreta. A combination of these two risk factors yielded the best performance with AUC of 0.880, sensitivity of 92% and specificity of 74%.

Placenta percreta is suggested when the villous tissue penetrates through the entire uterine wall, breaching the serosa and invading into surrounding organs [20]. Myometrium invasion increases the risk of postpartum hemorrhage, urologic injury and hysterectomy, when bladder invasion is present, maternal and neonatal mortality rates increased to 9.5% and 24%, respectively [21]. Despite various signs of PAS been suggested by SAR, the diagnosis of placenta percreta remains difficult [5]. On the one hand, not all accreta lesions presented with the typical morphological findings from MRI images; on the other hand, adherent and invasive placentation usually co-exist in the same bed and may further evolve with advancing gestation, resulting in a failure of accurately assessing the depth of myometrial invasion using one sign or a combination of several signs [22, 23].

Although T2 dark bands, placental heterogeneity, focal exophytic mass, abnormal vascularization of the placental bed, myometrial interruption, bladder tenting and parametrial vessel sign were associated with placenta percreta from our study, focal exophytic mass was the independent risk factor from the above MRI features for predicting placenta percreta. Focal exophytic mass usually is located toward the bladder or laterally toward the parametrium and is very specific for placenta percreta [24, 25]. The invasion of placental percreta can be limited to the uterine serosa, or outside the uterus to the bladder, when placental tissue involves the entire myometrial thickness abutting the bladder serosa, with a pressure effect or some nodularity of the bladder wall, the diagnosis of placenta percreta could be made [25].

IVIM is based on the conception that the distribution of water molecule in a voxel can be described using two compartments, the water molecular diffusion and blood microcirculation in the capillary network. In placental imaging, *f* is likely to represent the moving blood volume fraction compared with the total voxel volume, D* represents the movement of blood in the intervillous spaces and in the fetal capillaries within the villi and *D* represents the diffusion motion of pure water molecules [26, 27]. At low *b* values, the perfusion component predominates and is characterized by *f* and D*, while at high *b* values, the diffusion effect predominates and is characterized by *D*. Invasion of the placental villi into the myometrium will lead to vasodilation of the radial and arcuate uterine vasculature and neovascularization in the accreta lesions [22, 28]. Thus, placental perfusion increased in PAS disorders. The increase in *D* indicated the less restricted extracellular space in PAS disorders.

Different from conventional DWI, DKI is a non-Gaussian model that is believed to quantify non-Gaussian diffusion arising from diffusion barriers including cell membranes and organelles, or other complex and restricted structures in tissue using high *b* values [29]. Therefore, DKI may better describe the complicated water diffusivity in living tissues. MD is the corrected apparent diffusion coefficient (ADC) that accounts for non-Gaussian water diffusion and is analogous to the true water diffusion coefficient, *D* from IVIM, so MD and *D* were both significantly higher in patients with PAS disorders. In patients with placenta percreta, *f*, D* and MD were significantly higher than those in patients without PAS disorders, suggesting marked increase in placental vascularization and passive water molecular movement in percreta lesions.

In our study, D* remained as an independent risk factors from DWI in predicting placenta percreta, reflecting the prominent increase in microcirculatory perfusion in the capillary network in the placenta percreta. Chen et al.’ study showed a focal outward placental bulge with distorted outline of the uterus and bridging vessels running perpendicularly across through the focal bulging placenta, and serosal layer had 100% specificity in diagnosing placenta percreta [30]. Our results showing D* and focal exophytic mass the independent risk factors for placenta percreta may imply these bridging vessels are associated with increased microcirculatory perfusion in focal exophytic mass in placenta percreta. D* had moderate sensitivity and specificity, while focal exophytic mass had high specificity but low sensitivity. We further combined the two risk factors for predicting placenta percreta, resulting an AUC of 0.880, sensitivity of 92%, and specificity of 74%.

In PAS disorders, the placenta changed in both function and morphology. Morphological changes usually had accompanying functional changes including perfusion and diffusion. A comprehensive evaluation of the placenta can lead to more accurate prenatal diagnosis. Our findings about placenta percreta suggested when the chorionic villi penetrated through the myometrium to the uterine serosa or beyond the uterine serosa, the microcirculatory perfusion in the capillary network would increase accordingly. A combination of D* and focal exophytic mass can be used to identify cases at higher risk of placenta percreta to plan an appropriate surgical management.

This study had some limitations. First, it was retrospective, with a small sample size, and we had only 13 patients of placenta percreta, limiting the power of statistical analysis; thus, future studies with larger sample sizes, including more patients with placenta percreta, are needed. Second, the ROI delineation was performed by one radiologist. We did not calculate the intraclass correlation coefficient (ICC) between different readers. Our previous studies about PAS disorders using IVIM and DKI confirmed the high reproducibility of the ROI measurement [10, 11]. We believe that the recognition of MRI features of PAS requires experience; therefore, the radiologists evaluating MRI images in our study were experienced in obstetrical imaging. Third, we did not perform a comparison with US findings. Our patients were referred on the basis of suspicion for PAS from an uncertain US result, and some of US images were not available second to the tertiary referral nature of our practice.

Our study is the first one that tries to differentiate placenta percreta using both morphological features from MRI and functional parameters from DWI. Our results showed D* and focal exophytic mass were independently associated with placenta percreta. A combination of D* and focal exophytic mass can be used to differentiate placenta percreta, thus allowing for multidisciplinary care, planned preterm delivery, appropriate treatment options and improving patient prognosis.

## Data Availability

The datasets generated during and analyzed during the current study are not publicly available due to PACS system regulated by Sichuan Provincial People’s Hospital but are available from the corresponding author on reasonable request.
